# Switching from warfarin to direct-acting oral anticoagulants: it is time to move forward!

**DOI:** 10.1186/s43044-022-00259-9

**Published:** 2022-03-28

**Authors:** Mahmoud Abdelnabi, Juthipong Benjanuwattra, Osama Okasha, Abdallah Almaghraby, Yehia Saleh, Fady Gerges

**Affiliations:** 1 Internal Medicine Department, Texas Tech University Health Sciences Center, Lubbock, TX USA; 2 Cardiology and Angiology Unit, Clinical and Experimental Internal Medicine Department, Alexandria University, Alexandria, Egypt; 3 Internal Medicine Department, University of Missouri-Kansas City, Kansas City, MO USA; 4 Cardiology Department, Faculty of Medicine, Alexandria University, Alexandria, Egypt; 5 Cardiology Department, Houston Methodist DeBakey Heart & Vascular Center, Houston, TX USA; 6 Department of Cardiovascular Science, Mediclinic Al Jowhara Hospital, Al Ain, UAE

**Keywords:** Vitamin K antagonists, Warfarin, Direct-acting oral anticoagulants, Thromboembolism, Bleeding

## Abstract

Oral vitamin K antagonists (VKAs), warfarin, have been in routine clinical use for almost 70 years for various cardiovascular conditions. Direct-Acting Oral Anticoagulants (DOACs) have emerged as competitive alternatives for VKAs to prevent stroke in patients with non-valvular atrial fibrillation (AF) and have become the preferred choice in several clinical indications for anticoagulation. Recent guidelines have limited the use of DOACs to patients with non-valvular AF to reduce the risk of cardioembolic complications and to treat venous thromboembolism (VTE). Although emerging evidence is suggestive of its high efficacy, there was a lack of data to support DOACs safety profile in patients with mechanical valve prosthesis, intracardiac thrombi, or other conditions such as cardiac device implantation or catheter ablation. Therefore, several clinical trials have been conducted to assess the beneficial effects of using DOACs, instead of VKAs, for various non-guideline-approved indications. This review aimed to discuss the current guideline-approved indications for DOACs, advantages, and limitations of DOACs use in various clinical indications highlighting the potential emerging indications and remaining challenges for DOACs use. Several considerations are in favour of switching from warfarin to DOACs including superior efficacy, better adverse effect profile, fewer drug-drug interactions, and they do not require frequent international normalized ratio (INR) monitoring. Large randomized controlled trials are required to determine the safety and efficacy of their use in various clinical indications.

## Background

**Fig. 1:**
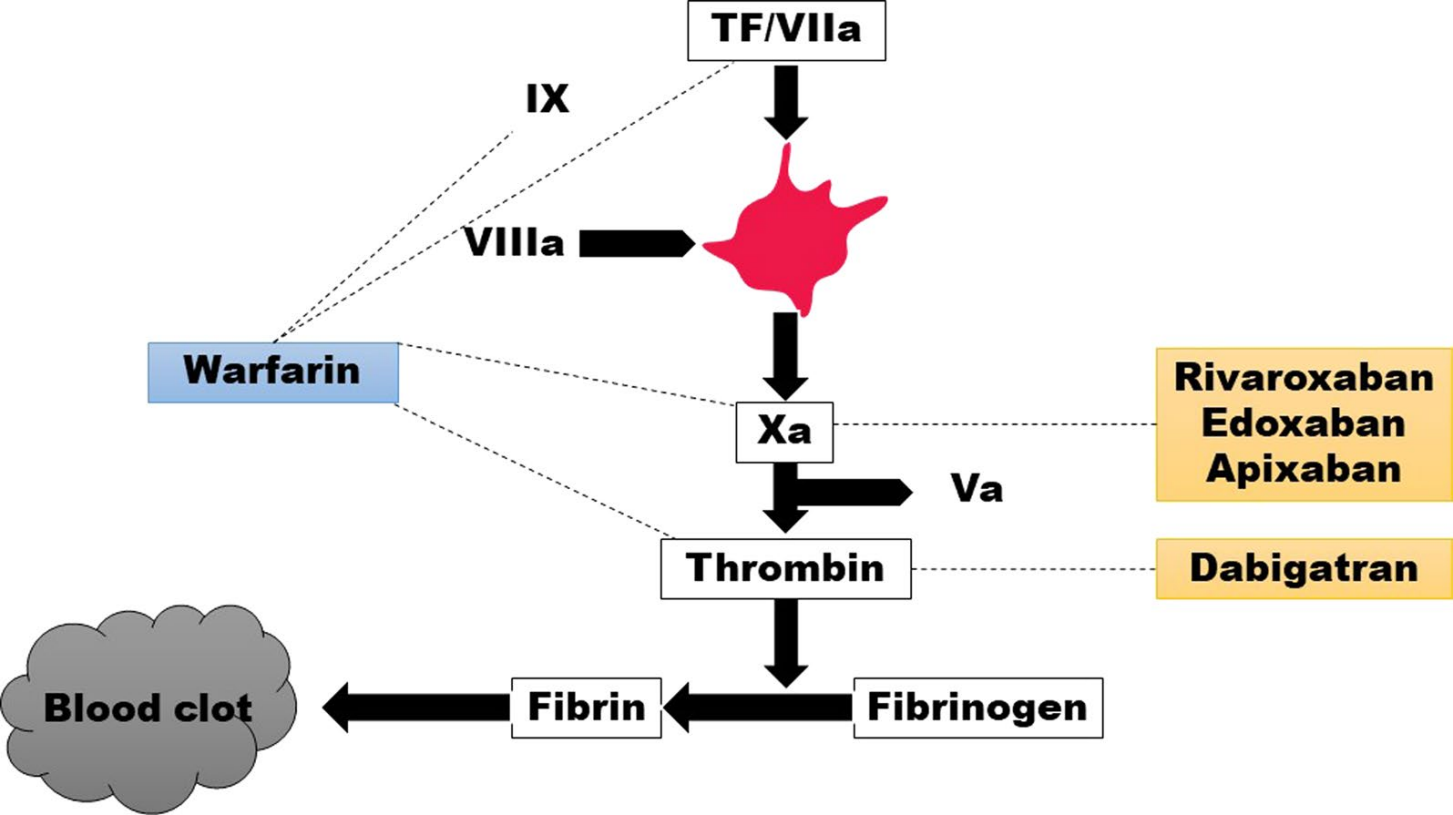
Sites of action of different anticoagulants

Warfarin was first clinically approved for human use in 1954 and, since then, it has been the most widely used anticoagulant worldwide prescribed for various cardiovascular indications [1]. It acts by inhibiting the vitamin K-dependent clotting factors (II, VII, IX, and X), as well as the natural anticoagulant factors (protein C and S) [1]. Currently, warfarin is commonly used in patients with non-valvular atrial fibrillation (AF) according to the calculated CHA_2_DS_2_-VASc score and in patients with valvular heart diseases (VHD) with AF due to the overwhelming evidence of its effectiveness in the prevention of cardioembolic strokes in these patients [2, 3]. In addition, it is also indicated in the treatment of deep venous thrombosis (DVT) and pulmonary embolism (PE) [4]. Despite its benefit, several drawbacks of warfarin are widely recognized such as narrow therapeutic range, slow onset and offset of action causing difficulty to manage during peri-invasive procedures, and multiple drug and food interactions. Warfarin is also less preferred by patients due to fear of side effects and complexity of management as it requires regular monitoring [1]. It goes without a doubt managing patients commencing warfarin requires a multi-disciplinary and multi-functional approach. Patient education should be an important component, although surprisingly, little attention has been paid to this [5]. A recent analysis of 6454 patients with AF taking warfarin showed that almost 50% of the time, the INR was outside the target range of 2–3 [6], leading to a higher risk of bleeding and thrombotic complications [7]. The major adverse effect associated with warfarin is bleeding. Major and fatal bleeding events occur at rates of 7.2 and 1.3 per 100 patient-years, respectively, according to a meta-analysis of 33 studies [8]. These limitations set a compelling demand for newer alternatives with better safety profiles. Direct-Acting Oral Anticoagulants (DOACs) are designed to overcome the drawbacks of warfarin. DOACs work by either direct inhibition of factor Xa (apixaban, edoxaban, and rivaroxaban) or thrombin (dabigatran) (Sites of action of different anticoagulants are summarized in Fig. 1) [9]. Unlike warfarin, DOACs have a rapid onset of action, shorter half-life, and more predictable therapeutic effects (Table 1) [10]. DOACs have been the patient’s preference over warfarin as they do not require routine monitoring, have fewer drug-drug interactions, and are not limited by the restriction of Vitamin K-containing food [9].

**Table 1:** Pharmacokinetic characteristics of NOACs compared to Warfarin

Characteristics	Warfarin	Apixaban	Dabigatran	Rivaroxaban	Edoxaban
Bioavailability	> 95%	~ 50% for doses up to 10 mg	~ 7%	> 80% for 10 mg dose (regardless of food intake) and 20 mg dose (taken with food); 66% for 20 mg dose (fasting)	~ 62% for 60 mg dose
Time to peak activity	24–36 h	3–4 h	0.5–2 h	2–4 h	1–2 h
Half-life	20–60 h	~ 12 h	11–14 h	5–9 h (young individuals); 11–13 h (elder individuals)	6–11 h
Dosing frequency in AF	Once daily	Twice daily	Twice daily	Once daily	Once daily
Drug interactions	Numerous drugs including substrates of CYP2C9, CYP3A4, and CYP1A2; various foods	Strong inhibitor/inducer of both CYP3A4 and P-gp	Strong P-gp inhibitor and inducer	Strong inhibitor of both CYP3A4 and P-gp; strong CYP3A4 inducer	Strong P-gp inhibitor
Renal elimination	< 1%	~ 27%	85%	66% of the dose undergoes metabolic degradation then 50% eliminated renally and 50% eliminated via hepatobiliary route. Other 33% undergoes direct renal excretion	50%

AF, atrial fibrillation; CYP, cytochrome P450; P-gp, P-glycoprotein

## Current clinical indications of DOACs

### DOACs in non-valvular atrial fibrillation

Several studies compared DOACs to warfarin for stroke prevention or systemic thromboembolism in patients with non-valvular AF. According to the ROCKET-AF trial, rivaroxaban was found to be non-inferior to warfarin in preventing stroke or systemic embolism in patients with non-valvular AF [11], whereas apixaban was superior to warfarin as revealed in the ARISTOTLE trial [12]. RE-LY trial showed non-inferiority when the reduced dose of dabigatran (110 mg twice daily) was used, while the full dose (150 mg twice daily) was superior to warfarin in preventing stroke and systemic embolism with similar rates of major bleeding [13]. All DOACs have been tested in large randomized prospective trials and resulted in documented efficacy and safety of the respective agent. Testing of different doses, however, was carried out differently. In ARISTOTLE (apixaban) and ROCKET-AF (rivaroxaban), patients received a single dose of 5 mg twice daily and 20 mg once daily, respectively, which were reduced in the presence of predefined patient characteristics [11, 12]. In contrast, in RE-LY (with dabigatran) and ENGAGE-AF (with edoxaban), both lower and higher doses were tested in fully powered patient cohorts [11–14]. Based on the overall clinical benefits of DOACs over VKAs, the European society of cardiology (ESC) and American college of cardiology (ACC) guidelines demonstrated a preference for DOACs over warfarin for stroke and systemic thromboembolism in non-valvular AF patients, especially if recently initiated. (Class I recommendation, level of evidence (LOE) A).

Several meta-analyses confirmed the safety and efficacy of DOACs over warfarin in real-world data of non-valvular AF. Deitelzweig et al., conducted a systematic review and network meta-analysis of real-world studies among non-valvular AF patients comparing major bleeding risk on DOACs or warfarin. The study concluded that in comparison to warfarin, DOACs were associated with a lower or same risk of major bleeding. Dabigatran had a significantly lower risk of major bleeding compared to warfarin and rivaroxaban, while apixaban had the lowest risk of major bleeding compared to other DOACs. [15] Waranugraha et al. aimed to assess the efficacy and safety of DOACs in a meta-analysis that involved 34 real-world studies including 2,287,288 non-valvular AF patients. The study concluded that DOACs were more effective in stroke reduction and all-cause mortality. Moreover, DOACs had a significantly lower major and intracranial bleeding risk. [16]

### DOACs in valvular atrial fibrillation

Compared to non-valvular AF, the risk of systemic thromboembolism, and stroke is even higher among AF patients with VHD. However, anticoagulation management strategies for this group of patients have been less studied by randomized trials, which mainly focused on non-valvular AF [17]. The first practical guide on the use of the DOACs in the management of non-valvular AF was suggested in 2013 [3]. Updates by the European Heart Rhythm Association (EHRA) were added in 2018 [18]. The recommendations for DOACs, rather than non-valvular AF, have been reviewed by Heidbuchel et al. [19]. The term valvular AF excluded AF patients with mechanical prosthetic valves and moderate to severe mitral stenosis which was excluded from all DOACs trials.

The following are proposed indications for the use of DOACs [19]:

- Mild to moderate native valve disease.
- Severe aortic stenosis, but limited data as most patients go to intervention.
- Bioprosthetic valves except for the first 3 months post-operative.
- Mitral valve repair except for the first 3–6 months post-operative.
- Percutaneous transluminal aortic valvuloplasty (PTAV); Transcatheter aortic valve implantation (TAVI) (limited prospective data)

Recently, a functional classification of AF patients with valvular heart diseases in relation to oral anticoagulation use was proposed. Type I VHD included moderate-severe rheumatic mitral stenosis and prosthetic valves (VHD requiring anticoagulation with VKAs, while type II included VHD other than type I requiring anticoagulation with VKAs or DOACs [20].

Further updates in ESC guidelines for VHD and AF management regarding the current approved indications of DOACS in stroke prevention in VHD patients eligible for anticoagulation. DOACs are preferred to VKAs in patients with aortic stenosis, aortic and mitral regurgitation (class I indication, LOE A), while DOACs were contraindicated in prosthetic mechanical valve and not recommended in moderate to severe mitral stenosis (class III indication, LOE B, C, respectively) [17, 21]

### DOACs in non-valvular atrial fibrillation with acute coronary syndromes

Dual antiplatelet therapy prevents stent thrombosis and is indicated for secondary prevention in those with acute coronary syndromes (ACS). On the contrary, oral anticoagulants are indicated in atrial fibrillation to prevent thromboembolism. According to the ESC guidelines published in 2020 [22], recent evidence suggests that DOACs have a superior safety profile compared to VKAs in ACS patients undergoing percutaneous coronary intervention (PCI) with AF without mechanical prostheses or moderate-to-severe mitral stenosis. Giving with P2Y12 inhibitor, apixaban 5 mg twice daily was associated with lower incidence of bleeding and the composite of death or hospitalization than VKAs in those with recent ACS or PCI [23]. Edoxaban 60 mg once daily with a P2Y12 inhibitor for 12 months was non-inferior in terms of bleeding events to a triple therapy regimen consisting of VKAs, P2Y12 inhibitor, and aspirin in patients with successful PCI. The incidence of thromboembolism and cardiovascular death was also not different [24]. In contrast, the risk of bleeding from dabigatran 110 or 150 mg twice daily with P2Y12 inhibitor was lower than triple therapy with VKA in those who had undergone PCI and was non-inferior in preventing thromboembolic events [25]. Lastly, rivaroxaban 15 mg once daily with clopidogrel or low-dose rivaroxaban 2.5 mg twice daily with aspirin and clopidogrel was associated with lower bleeding events, hospitalization, and all-cause death but not cardiovascular death or thromboembolic events compared to triple therapy with warfarin [26]. The recommendation has been made by the ESC in AF patients with non-ST-elevation myocardial infarction (NSTEMI) undergoing PCI to receive triple therapy with DOACs plus dual antiplatelet during hospitalization followed by DOACs plus antiplatelet for 12 months then DOACs monotherapy thereafter [22]. In those with high ischemic risk, it is recommended to use DOACs plus dual antiplatelet for one month followed by DOACs plus antiplatelet until 12 months then monotherapy with DOACs [22]. Guidelines from the ACC/American Heart Association (AHA)/Heart Rhythm Society (HRS) recommend the use of clopidogrel with either dabigatran 150 mg twice daily or rivaroxaban 15 mg daily in those with AF complicating ACS with CHA_2_DS_2_-VASc score of 2 or greater and also suggests that the use of triple therapy should be limited to only 4–6 weeks following PCI [27].

### DOACs in venous thromboembolism

The most recent guidelines on the management of VTE from the American College of Chest Physicians (ACCP) released in 2016 [28] differ notably from previous recommendations in 2012 [29]. The earlier guidelines recommended the use of VKAs over low-molecular-weight heparin (LMWH) in patients without cancer and LMWH over VKAs in patients with cancer [29]. VKAs were recommended over DOACs (dabigatran and rivaroxaban) in both groups of patients given the existing evidence at that time [29]. The 2016 ACCP guidelines take into consideration the large body of evidence gathered in recent years supporting the use of DOACs in the treatment of acute VTE [28]. Based on these updated guidelines, DOACs are currently recommended over VKAs during the first 3 months of anticoagulation therapy for patients with proximal deep venous thrombosis (DVT) of the leg or PE in those without cancer, whereas LMWH is still the anticoagulant of choice in cancer patients [28]. Guidelines from ESC that were published in 2019 also included recommendations for the extended treatment duration for VTE [30]. These guidelines identified patients with cancer and those who have experienced an unprovoked proximal DVT or pulmonary embolism (PE) who are at low risk of bleeding as candidates for indefinite treatment. Although LMWH is recommended for the first 6 months in cancer patients with PE (Class II A, LOE A), the guidelines also comment on the use of DOACs with a recommendation (Class II A, LOE B) to consider dabigatran, rivaroxaban, or apixaban over VKA in patients who require extended anticoagulation therapy [30]. DOACs could be a much more convenient option in patients with cancer, compared with LMWH, as they are orally administered in a fixed-dose regimen. However, only 3–9% of patients included in phase III studies with DOACs for the treatment of VTE had concomitant cancer [31–35]. According to the latest 2019 ESC Guidelines for the diagnosis and management of acute pulmonary embolism and recommendations for the extended treatment of VTE, edoxaban (Class II A, LOE B) and rivaroxaban (Class II A, LOE C) should be considered as an alternative to LMWH in patients with and without gastrointestinal cancer, respectively [36, 37].

### DOACs in Pregnancy

There are extremely limited data on the safety of DOACs use during pregnancy [38]. All major NOAC trials excluded patients who were pregnant. Dabigatran, rivaroxaban, and Edoxaban are classified by the Food and Drug Administration (FDA) as a pregnancy class C: “risk cannot be ruled out”. Although apixaban is classified as a pregnancy class B: “animal reproduction studies have failed to demonstrate a risk to the fetus and there are no adequate and well‐controlled studies in pregnant women”, there are no clinical trials to justify the efficacy and safety of DOACs in pregnancy [39].

### DOACs in acute medically ill patients

Betrixaban is an extremely potent factor Xa inhibitor with minimal renal clearance and minimal hepatic metabolism [40]. Betrixaban is the only FDA-approved DOACs for extended-duration prophylaxis for VTE in acute medically ill patients (Direct comparisons of betrixaban and the other direct oral anticoagulants can be found in Table 2) [41–43].

**Table 2:** Comparison between oral factor Xa inhibitors

	Apixaban	Rivaroxaban	Betrixaban
Onset	3–4 h	Rapid	Rapid
Protein binding	87%	92–95%	60%
Metabolism	Hepatic; predominantly 3A4 Substrate of P-gp	Hepatic; predominantly 3A4 Substrate of P-gp	Minimal hepatic metabolism Substrate of P-gp
Bioavailability	50%	66–100%	34%
Elimination half-life	2.5 mg ~ 8 h 5 mg ~ 15 h	5–9 h	37 h
Excretion	Urine ~ 27% as parent drug Feces ~ 25% as metabolites	Urine 36% as unchanged drug	Biliary system ~ 85% as unchanged drug Urine < 8%

P-gp, P-glycoprotein

### DOACs in antiphospholipid syndrome

Previous reports were inconclusive regarding the benefit or harm of DOACs in anti-phospholipid syndrome (APS) [44, 45]. In 2018, a multicentre, randomized, open-label study evaluating the efficacy and safety of rivaroxaban, compared to warfarin, in high-risk patients with antiphospholipid syndrome revealed unfavourable results associated with the use of rivaroxaban, resulting in early termination of the trial [46]. Rivaroxaban was associated with increased thromboembolic events in 12% of patients, while no such complication occurred in the warfarin group. Bleeding was also reported in 7% and 3% of the rivaroxaban and warfarin groups, respectively [46]. With current evidence, VKAs remain a better option than DOACs for patients with antiphospholipid syndrome.

## Eligibility for DOACs therapy in other clinical indications

DOACs have been well-approved for stroke prevention in non-valvular AF. Strictly, the term ‘non-valvular AF’ refers to AF in the absence of a mechanical prosthetic heart valve or moderate to severe mitral stenosis (usually of rheumatic origin) [2, 47, 48], as patients with these conditions were excluded from trials. Other recent indications where numerous case reports and studies successfully demonstrated the eligibility for using DOACs are:

### DOACs in intracardiac thrombi

The utilizations of apixaban, dabigatran, and rivaroxaban in the setting of intracardiac thrombi have been reported in multiple case studies and clinical trials. The use of apixaban 5 mg twice daily instead of warfarin, due to previous labile INR and gastrointestinal bleeding, in a case of 55 years old man with left ventricular (LV) apical thrombus was associated with dramatic resolution of thrombus after one month [49]. In a 60 years old woman with LV thrombus secondary to hypertrophic cardiomyopathy and AF, apixaban 5 mg twice daily also led to resolution of a 2 × 3 cm thrombus at one-month follow-up [50].

Dabigatran 110 mg twice daily was associated with a complete thrombus resolution in a patient with hypertrophic cardiomyopathy and AF who previously had labile international normalized ratio (INR) warranting discontinuation of Warfarin [51], and in a patient with acute ischemic stroke due to LV thrombus [52]. Rivaroxaban 15 mg once daily led to a complete thrombus resolution after one month in a 78-year-old man with non-valvular AF and heart failure who had a previous difficulty in achieving therapeutic INR levels with Warfarin [53]. A longer duration of treatment was needed in the case reported by Seecheran et al., for treatment of a 2.5 × 1.5 cm LV apical thrombus in a patient with acute ST-elevation myocardial infarction [54]. Abdelnabi et al., studied the effects of rivaroxaban in a case series of 8 patients presented with ACS and LV thrombus [55]. It was concluded that rivaroxaban, in conjunction with dual antiplatelet therapy, was effective and safe in the treatment of LV thrombi without increasing the bleeding risk [55]. The NO-LVT trial was the first randomized controlled trial (RCT) to compare rivaroxaban to warfarin in 79 patients with LV thrombus The study concluded that rivaroxaban was non-inferior to warfarin in thrombus dissolution and had even faster thrombus resolution at 1 month. Regarding safety, major bleeding occurred in 2 patients (5.1%) in the rivaroxaban group and in 6 patients (15%) in the warfarin group while composite thromboembolic events were zero in the rivaroxaban group and 6 patients (15%) in the warfarin group [56]. Alcalai et al., randomized 35 patients with LV thrombi after acute myocardial infarction (MI) to apixaban or warfarin concluding that apixaban was non-inferior to warfarin for LV thrombi treatment post-acute MI after 3 months of anticoagulation therapy [57].

Several meta-analyses studied the safety and efficacy of DOACs over warfarin in real-world data of patients with LV thrombi. Saleh et al., in a meta-analysis comparing rivaroxaban to warfarin in patients with LV thrombi concluded that rivaroxaban had similar rates of systemic thromboembolism, and bleeding. Moreover, rivaroxaban had higher rates of thrombus resolution, but it did not reach statistical significance [58].

### DOACs in left atrial and left atrial appendage thrombi

The left atrial appendage (LAA) is the most common location for thrombus formation in patients with AF [59]. Left atrial (LA) and LAA thrombus formation are attributed to many predisposing factors such as CHA2DS2-VASc score that is equal to or more than 2, increased LA/LAA volume, and morphological variances [59]. Prevention of formation of these thrombi and their embolization by using DOACs is well established in the guidelines. However, commencing DOACs in the treatment of already formed thrombi is still under research. Numerous case reports and trials for successful resolution of LA and LAA thrombi after using DOACs are available. Dabigatran, at a dose of 150 mg twice daily for approximately 7 weeks, led to a complete resolution of 0.8 × 0.8 cm LAA thrombus in a 59-year-old woman who was previously commenced on warfarin [60]. However, a failure of low-dose dabigatran in preventing thrombus formation in a patient with AF was reported by Koyama et al. [61]. Apixaban 5 mg twice daily was initiated, and a complete LAA thrombus resolution was achieved after 8 weeks. A failure of Dabigatran in preventing thrombus formation might be explained by a lower dose used in this patient; however, the exact mechanism remains inconclusive [61]. Both rivaroxaban and apixaban seem to be promising agents in preventing and dissolving LA/LAA thrombus. Rivaroxaban 20 mg daily was associated with a complete thrombus resolution after 5 weeks in a 49 years old woman with severe valvular AF [62]. In a case series of 12 patients with AF and LAA thrombus, rivaroxaban 20 mg once daily for 3 weeks was associated with a total resolution of LAA thrombi in 11 patients without increased risk of bleeding or systemic thromboembolization [63]. A prospective study using a standard dose of rivaroxaban 20 mg daily in patients with LA/LAA thrombus secondary to non-valvular AF or atrial flutter revealed a comparable thrombus resolution to those treated with heparin/VKAs [64]. In 2020, a recently published RIVA-TWICE study comprising 15 patients with AF and persistent LAA thrombus despite taking rivaroxaban 20 mg daily showed that rivaroxaban 15 mg twice daily was associated with higher activities of anti-Xa factor, leading to a complete thrombus resolution in 46.7% of patients after 8 weeks [65]. A complete resolution of thrombus after treatment with apixaban 5 mg twice daily was, for the first time, reported in a 72-year-old male patient with persistent AF [66]. Another successful LAA thrombus resolution by Apixaban was reported in an 84-year-old woman with heart failure and AF after failure to achieve therapeutic INR levels with warfarin [67]. Similarly, in the EMANATE trial, thrombus resolution rate in patients with AF was similar in those treated with apixaban (52%) as with conventional heparin/VKAs therapy (56%) [68]. In congregate, these data indicate that DOACs may represent another treatment option for LA/LAA thrombus (best data available for rivaroxaban and apixaban), particularly in patients for whom VKAs is not well-tolerated or therapeutic INR levels cannot be achieved.

### DOACs in post-transcatheter aortic valve implantation

Stroke is a devastating embolic complication occurring in up to 7% of patients within the first year following TAVI or surgical valve replacement [69]. The risk of transcatheter aortic valve thrombosis is highest within the first few months; however, it can occur at a variable time after TAVI [70–72]. The appropriate antithrombotic regimen following TAVI remains a matter of debate, with US and European guidelines offering an array of weak recommendations. According to the most recent ESC guidelines, dual antiplatelet therapy (DAPT) should be considered for the first 3 to 6 months after TAVI (Class II A, LOE C), while oral anticoagulation should only be considered for patients with other indications for therapy, such as those with AF. Regarding the AHA/ACC guidelines, there is a class II B recommendation for at least 3 months of oral anticoagulation after TAVI in patients at low bleeding risk, in addition to DAPT during the first 6 months (class II B, LOE C) [73]. However, the recommendation of oral anticoagulation is largely based on VKAs. According to the study of 962 patients undergoing TAVI, although the 1-year risk of bleeding and all-cause mortality were not different between DOACs and VKAs, there was a higher rate of ischemic events in the DOACs group [74]. An RCT recently published in 2020 investigating the role of aspirin plus rivaroxaban 10 mg daily, compared to aspirin alone, after TAVI in 1644 patients revealed disappointing results showing that rivaroxaban was associated with a higher risk of thromboembolic events and death. Unfortunately, it is still unclear why rivaroxaban was associated with higher mortality [75]. With currently limited evidence, the decision to use DOACs for the sole purpose of thromboembolic prevention following TAVI remains questionable [73]. Several RCTs studying the impact of DOACs after TAVI have been investigated including the ENVISAGE-TAVI AF trial (edoxaban) and ATLANTIS trial (apixaban) [76, 77]. The results from the ATLANTIS trial suggested that apixaban is not recommended in those without indications for anticoagulation as there was higher non-cardiovascular mortality compared to antiplatelet alone. However, in patients requiring long-term anticoagulants, it was concluded that apixaban can be used instead of VKAs [76]. From the ENVISAGE-TAVI AF trial, edoxaban was found to be non-inferior to VKAs; however, bleeding events, particularly gastrointestinal bleeding, were higher in the edoxaban group [77].

### DOACs in prosthetic valves

Several trials were conducted to evaluate the efficacy and safety of DOACs in prosthetic valves, such as The RE-ALIGN study which was prematurely terminated due to safety concerns. The RE-ALIGN study concluded that in patients with mechanical mitral or aortic valves, dabigatran was not only less effective than warfarin for thromboembolic prevention but was also associated with an increased risk of bleeding; therefore, it should not be used in mechanical valve patients [78]. Since Dabigatran only inhibits thrombin, while thrombin, factor IX, X, and tissue factor-induced coagulation (factor VII) are inhibited by Warfarin, an overwhelming coagulation activation and subsequent thrombin formation may exceed dabigatran’s inhibitory capacity [78]. The DAWA trial which was a prospective pilot study evaluating the efficacy and safety of dabigatran versus warfarin in Patients with bioprosthetic mitral and/or aortic valve replacement and AF. It was terminated prematurely due to low enrollment; however, the ability to prevent intracardiac thrombus formation was similar between dabigatran and warfarin [79]. In contrast to dabigatran, the use of rivaroxaban in a pilot study of 7 patients for 90 days following mechanical mitral valve replacement was not associated with thromboembolic or bleeding events [80]. The result of this pilot study suggests that Rivaroxaban may be a feasible alternative to warfarin [80]. Additionally, the RIVER trial compared the safety and efficacy of rivaroxaban 20 mg daily to dose-adjusted warfarin in 1005 AF patients with bioprosthetic mitral valve. The primary outcome was composite of death, major cardiovascular events (stroke, transient ischemic attack, systemic embolism, valve thrombosis, or hospitalization for heart failure), or major bleeding at 12 months. The study concluded that rivaroxaban was non-inferior to warfarin in AF patients with mitral bioprosthetic valves. [81] Hong et al. compared the safety and efficacy of edoxaban to warfarin early after surgical bioprosthetic valve implantation (BPV) or valve repair. The primary outcome was a composite of death, symptomatic thromboembolic events, or asymptomatic intracardiac thrombosis concluding that edoxaban use was non-inferior to warfarin in thromboembolism prevention or major bleeding in the 1st three months after BPV implantation or valve repair [82]. Nevertheless, future large RCTs are warranted to further evaluate its safety and efficacy in prosthetic valves.

### DOACs in cardiac device implantation and catheter ablation

Many patients who undergo procedures for cardiovascular electronic device implantation have concomitant AF which is an indication for anticoagulation. During the peri-procedural period, the risk of complications such as pocket bleeding and hematoma must be taken into account while commencing anticoagulant [83]. In comparison to uninterrupted Warfarin, Jennings et al., have concluded that there was no difference in the bleeding incidence between uninterrupted dabigatran and warfarin during implantation of cardiovascular implantable electronic devices [83]. In agreement, a prospective study done by Rowley et al., on 25 patients undergoing cardiac device implantation under the cover of Dabigatran showed no thromboembolic or major bleeding complications within 30 days of operation [84]. Recently published RCTs also seem to confirm the safety and efficacy of uninterrupted DOACs in the setting of AF catheter ablation. The VENTURE-AF trial (*n* = 248), comparing uninterrupted rivaroxaban to uninterrupted VKAs, reported no major adverse outcomes within the first 30 days after the procedure with rivaroxaban, compared with 1 major bleeding, 1 ischemic stroke, and 1 vascular death in the warfarin group [85]. Another promising result was obtained from the RE-CIRCUIT trial (*n* = 704), in which uninterrupted dabigatran was associated with fewer major bleeding events compared with warfarin (5 versus 22 events, *P* < 0.001) during 8-week follow-up [86].

### DOACs in chronic kidney disease

DOACs appear to be safe and effective in chronic kidney disease (CKD) population. A study of 21,733 patients with CKD and non-valvular AF with CHA_2_DS_2_-VASc score of 2 or greater showed that DOACs are associated with lower bleeding risk and all-cause mortality with similar embolic stroke events compared to warfarin across all ranges of kidney function [87]. Data from a recent meta-analysis of patients with non-valvular AF or VTE also suggested a better efficacy of DOACs in preventing stroke, embolism, and VTE, compared to warfarin, in early-stage CKD. The efficacy is similar in CKD stage 4–5 and dialysis patients but DOACs are associated with a lower risk of bleeding in CKD stage 4–5 [88]. According to the subgroup analysis, apixaban exhibits superior efficacy and safety profiles compared to other factor Xa inhibitors [88].

### DOACs in liver disease

The dysregulation of homeostasis in cirrhosis is complex and involves both procoagulant and anticoagulant effects. Despite having prolonged INR and thrombocytopenia, cirrhotic patients have an elevated risk of VTE [89]. Evidence regarding the safety and efficacy of DOACs in chronic liver disease is limited. It is suggested that DOACs may be used with similar rates of major bleeding to warfarin in mild-to-moderate liver disease [90]. Guidelines from EHRA suggest that apixaban, dabigatran, edoxaban, but not rivaroxaban, may be used with caution in cirrhosis with Child–Pugh class B [18, 89]. DOACs are not recommended in severe cirrhosis with Child–Pugh class C as data are extremely scarce likely as most patients were excluded from the study due to high bleeding risk, particularly from esophageal varices [90].

### DOACs in the geriatric age group

Although there are no guidelines for DOACs use in elderly AF patients, recent studies investigated DOACs efficacy and safety in this age group. A meta-analysis that included five phase III RCT recruiting 28,137 patients (aged ≥ 75 years) concluded that in comparison to warfarin, DOACs had a lower risk of stroke, systemic thromboembolism, and major bleeding, with apixaban being the safest to be used for in stroke prevention for elderly AF patients [91]. A nationwide cohort study assessed thromboembolism risk and major bleeding associated with anticoagulation initiation among 30,401 AF patients aged ≥ 75 years. The study concluded that in comparison to warfarin, standard and reduced doses of DOACs were associated with similar risks of stroke/SE as warfarin and lower or similar risks of bleeding. [92] Polymeris et al., in a multi-centre prospective cohort study, investigated the safety and efficacy of DOACs in comparison to warfarin in AF patients aged ≥ 85 years after a recent stroke (< 3 months). The study concluded that DOACs were associated with a lower risk of the composite outcome of recurrent ischemic stroke, intracranial haemorrhage, and all-cause mortality in patients with AF and recent ischemic stroke, independent of age [93].

### Strategy for switching from Warfarin to DOACs

**Fig. 2:**
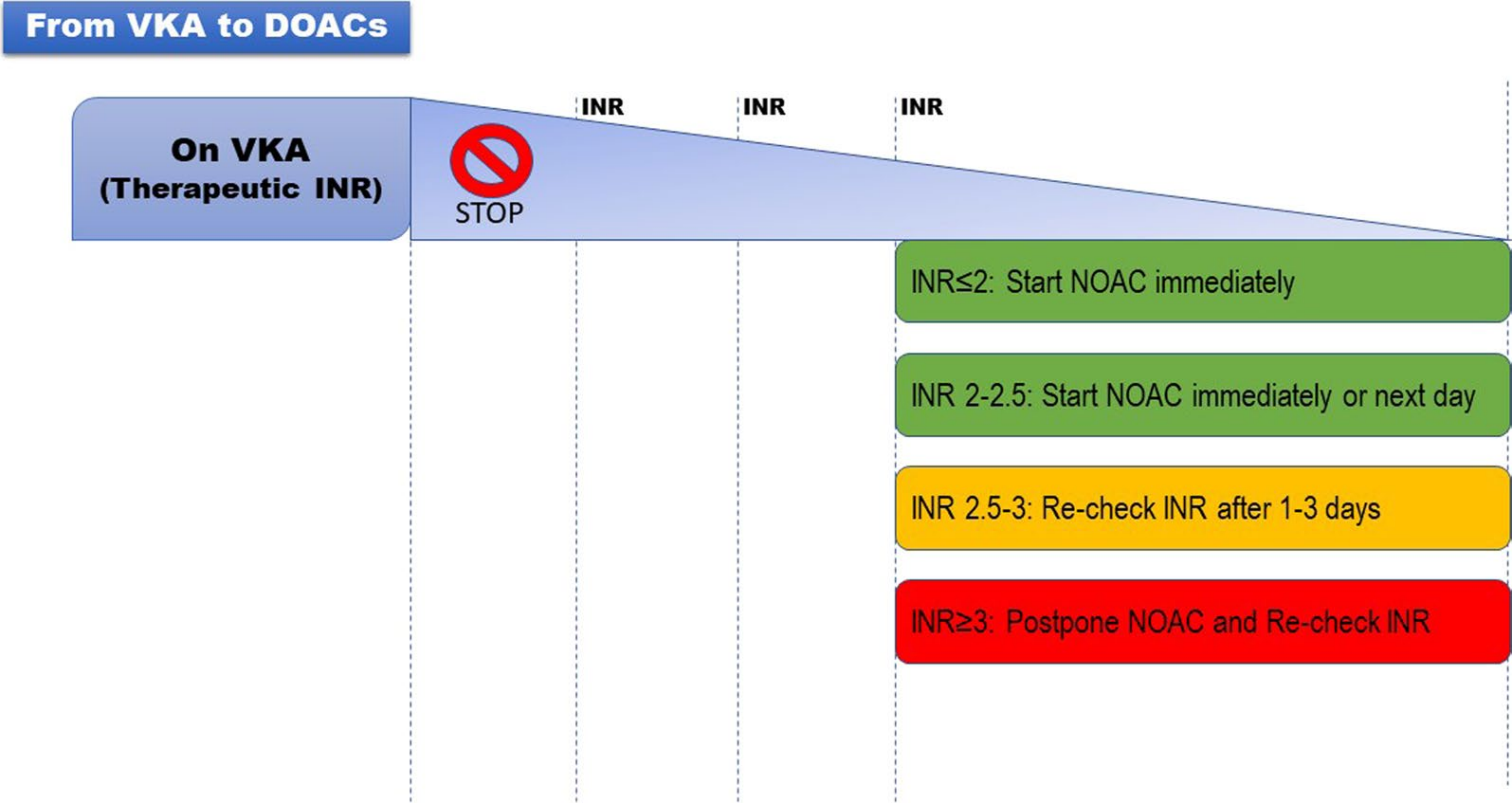
Switching from VKA to DOACs

**Fig. 3:**
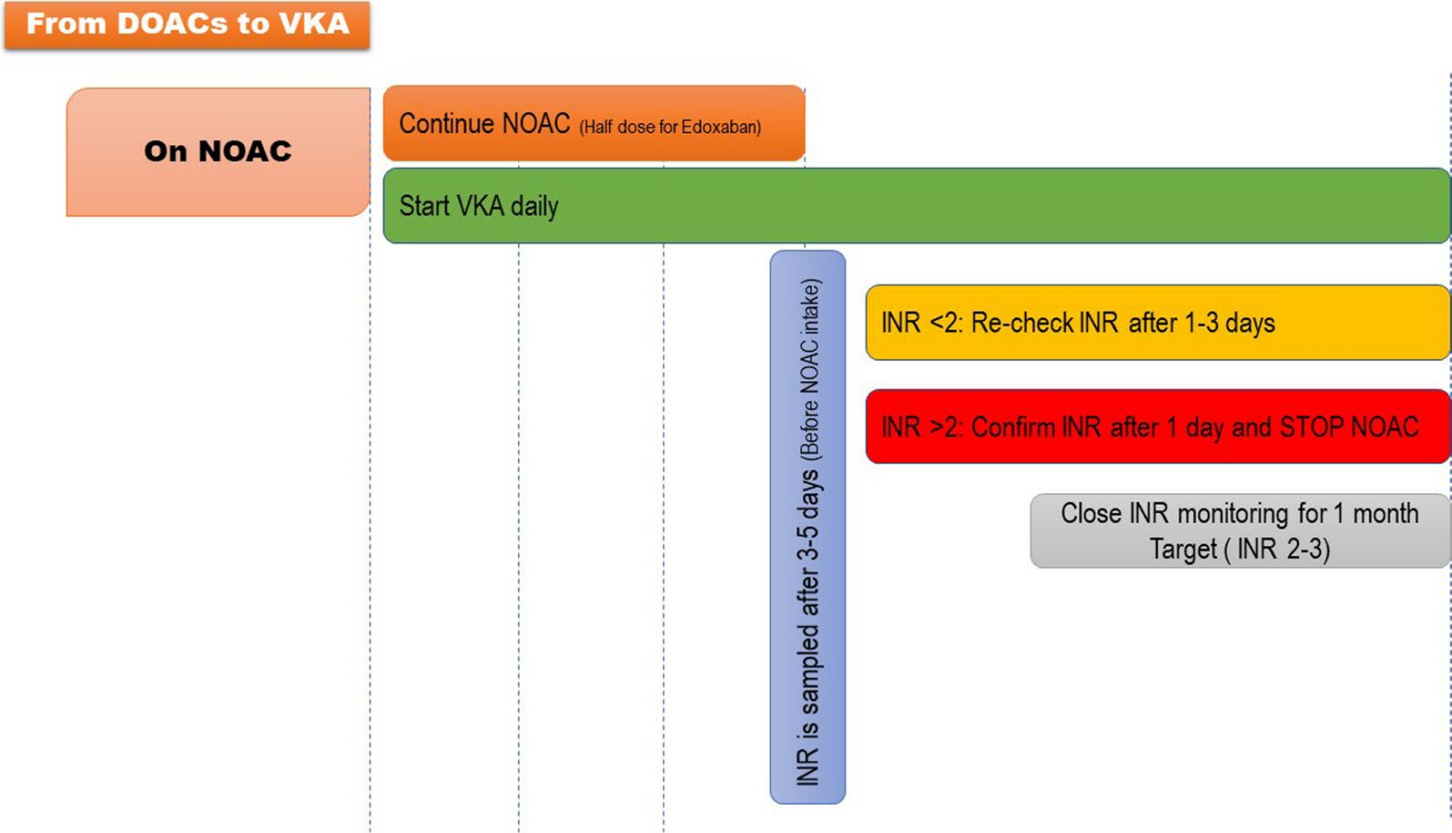
Switching from DOACs to VKA

It is safe to promptly initiate DOACs once the INR is ≤ 2.0. If the INR is 2.0–2.5, DOACs can also be started immediately or the following day. However, if the INR is > 2.5, it is recommended that the actual INR level and the half-life of Warfarin (36–48 h) be taken into consideration for estimating the appropriate starting time. According to the European guidelines, rivaroxaban, edoxaban, apixaban, and dabigatran can be initiated once the INR is ≤ 3, ≤ 2.5, and ≤ 2, respectively (Figs. 2, 3) [18].

### Challenges for DOACs use

**Fig. 4:**
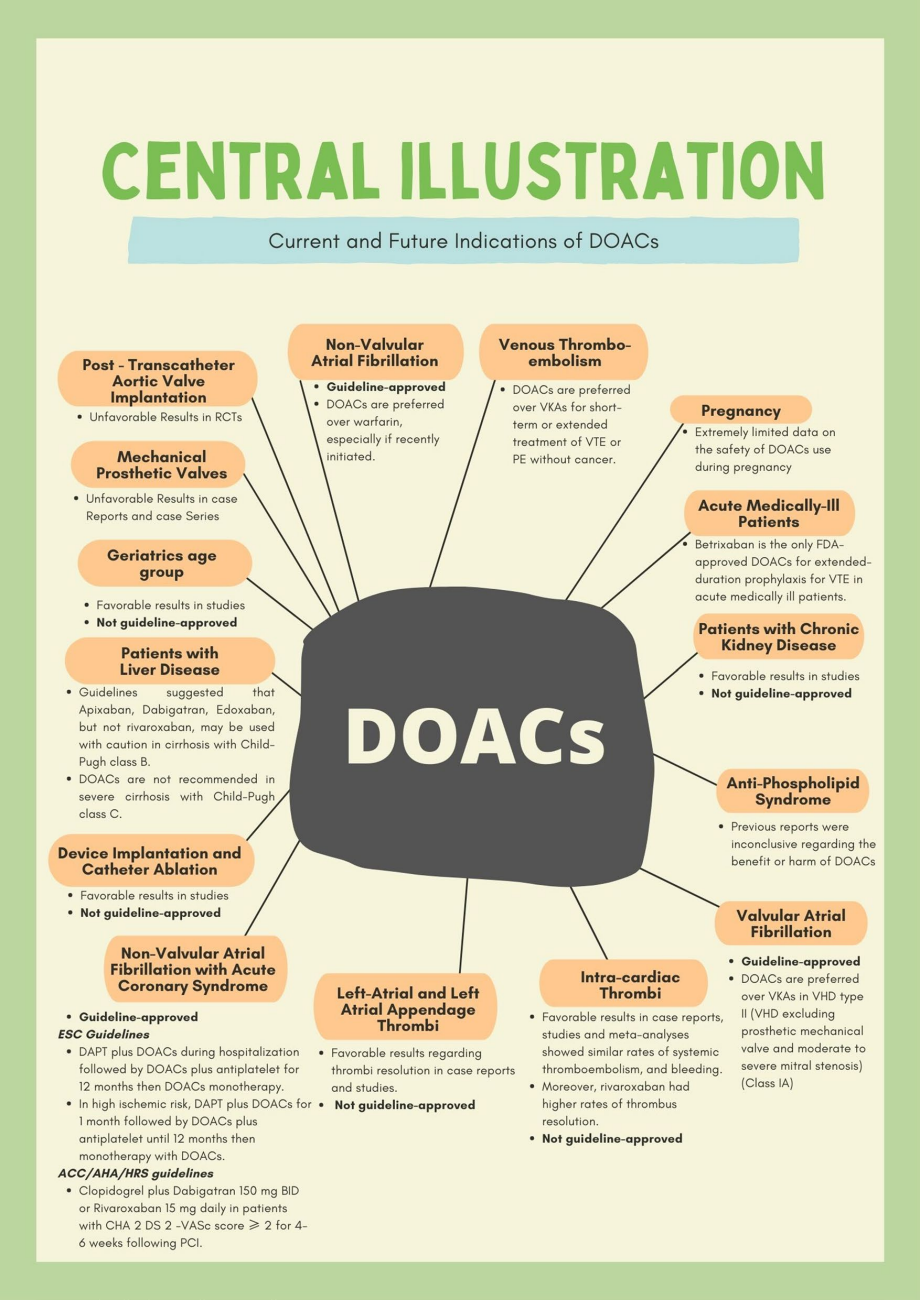
Central illustration showing guideline recommended indications and proposed other indications of DOACs

Despite its promising benefits, certain limitations are restricting the mass utilization of DOACs in healthcare systems. One of the major restrictions is the cost. Another concern is the presence of comorbidities including impaired renal function which interferes with the clearance of DOACs and may lead to an increased risk of bleeding [9]. The adherence to treatment may also be compromised with unmonitored anticoagulant therapy so meticulous dosing must be optimized in vulnerable patient populations such as the elderly [9]. Patients at the extreme weight spectrum (i.e. < 60 kg and > 150 kg) have been underrepresented in the clinical trials; hence, selecting the optimal dose of DOACs may be challenging in these individuals. Although an assessment of plasma trough levels may be helpful, further pharmacokinetic/dynamic studies in these populations would be beneficial [9]. Also, DOACs use in prosthetic valve patients are still questionable due to the limited data regarding their safety and efficacy [94]. Central illustration showing guideline recommended indications and proposed other indications in different patient groups is shown in Fig. 4

## Conclusions

DOACs are a great step forward in the field of anticoagulation. VKAs have been replaced by DOACs in the guidelines for various indications. Nevertheless, due to the lack of safety profile and extensive clinical studies, DOACs are not yet approved for many other indications. Future clinical trials are warranted to justify the utilization of DOACs in those settings. Therefore, the question still stands. Can those newcomers inherit the legacy of VKAs? Is it time to move forward with DOACs? or Warfarin will maintain its place for many years to come!

## Abbreviations

VKAs: Vitamin K antagonists
DOACs: Direct-acting oral anticoagulants
AF: Atrial fibrillation
VTE: Venous thromboembolism
INR: International normalized ratio
VHD: Valvular heart diseases
DVT: Deep vein thrombosis
PE: Pulmonary embolism
ESC: European Society of Cardiology
ACC: American College of Cardiology
EHRA: European Heart Rhythm Association
PTAV: Percutaneous transluminal aortic valvuloplasty
TAVI: Transcatheter aortic valve implantation
ACS: Acute coronary syndromes
PCI: Percutaneous coronary intervention
NSTEMI: Non-ST-elevation myocardial infarction
AHA: American Heart Association
HRS: Heart Rhythm Society
ACCP: American College of Clinical Pharmacology
LMWH: Low molecular weight heparin
FDA: Food and Drug Administration
APS: Antiphospholipid syndrome
LV: Left ventricle
RCT: Randomized controlled trial
LAA: Left atrial appendage
LA: Left atrium
DAPT: Dual antiplatelet therapy
AHA/ACC: American Heart Association/American College of Cardiology
BPV: Bioprosthetic valve
LOE: Level of evidence
MI: Myocardial infarction
CKD: Chronic kidney disease
